# Effects of Pre-Anthesis Drought, Heat and Their Combination on the Growth, Yield and Physiology of diverse Wheat (*Triticum aestivum* L.) Genotypes Varying in Sensitivity to Heat and drought stress

**DOI:** 10.1038/s41598-019-43477-z

**Published:** 2019-05-06

**Authors:** Mirza Faisal Qaseem, Rahmatullah Qureshi, Humaira Shaheen

**Affiliations:** 1 Department of Botany PMAS-Arid Agriculture University Rawalpindi Pakistan, Rawalpindi, Pakistan; 2 Department of Biosciences COMSATS University Islamabad, Park Road, Islamabad 45550, Pakistan, Islamabad, Pakistan

**Keywords:** Plant breeding, Plant physiology

## Abstract

Independent and combined drought and heat stress negatively affect wheat yield and physiology. The present study was aimed to quantify effects of Drought [D], heat [H] and combined heat and drought [HD] during reproductive stage on wheat yield and to identify physio-biochemical traits which were strongly associated with improved yield and tolerance of wheat under stressful conditions. One hundred and eight elite diverse wheat genotypes were exposed to [H], [D] and [HD] treatments from heading till maturity. Grain yield was reduced by 56.47%, 53.05% and 44.66% under [HD], [H] and [D] treatment, respectively. The [HD] treatment affects the grain yield by reducing metabolism and mobilization of reserves to developing grains and leaves. Disintegration of membrane structure, chlorophyll and protein molecules was higher under [H] stress than [D] stress while water status of genotypes and sink strength was more affected by [D] than [H] stress. Multivariate analysis showed a strong correlation of chlorophyll content before and after anthesis, water-soluble carbohydrates (WSC), proline content (PC) and all other studies agronomic and physiological traits with grain yield while days to anthesis (DTA) and days to maturity (DTM) were negatively associated with grain yield under stress showing advantage of early maturity during stress. Traits having a major contribution in the first two principal components under different stress treatments may lead to improved varieties with heat and drought stress tolerance. To best of over knowledge, the present study is the first detailed study which used physiological and biochemical traits to explain the variation in grain yield and related traits in diverse wheat germplasm.

## Introduction

Wheat is the staple food contributing 20% calories to world’s population, with total harvest area of 2.1 million km^2^ and global production of 700 million tonnes^1,2^. In rain fed areas wheat is ranked first as far as the area of cultivation is concerned and under irrigated conditions, it is ranked second after rice based on total cultivation area^3^. Due to its cultivation in rain fed conditions, it faces high temperature combined with irregular water supply (drought stress) during grain filling duration which results in yield reduction^4–7^. Major effects of high temperature include the reduction in the crop cycle, pollen abortion, kernel shrinkage, reduction in seed reserves, anther indehiscence and reduced development of the pollen tube, all these events result in reduced crop yield^2,8^. Furthermore, high temperature also affects some physiological processes, mainly impairment in photosynthesis and respiration, disintegration of chlorophyll, damage to photosystem II of the photosynthetic apparatus^6,9,10^. Drought stress is also detrimental to wheat growth some of major effects of drought on wheat include: reduction in leaf area, kernel abortion, reduced mobilization of reserves and reduced number of amyloplasts in grain^9,11^. The harmful effects of the dry spell on wheat yield may exacerbate while occurring with heat stress^6^. Simultaneous heat and drought stress initiate various processes like a decreased rate of photosynthesis coupled with abnormal respiration, closed stomata and high leaf temperature^12^. These effects may be synergistic, antagonistic or hypo-additive on yield or any other trait^6,13,14^. Furthermore, it is proven by various studies that impact of heat and drought stress on growth, yield and physiology varied among different crops. The effects of simultaneous heat and drought stress are studied in various crops including wheat (*Triticum aestivum* L.^15^), maize (*Zea mays* L.^16^), groundnut (*Arachis hypogaea* L.^17^) and others^18–20^. To best of our knowledge, the present study is first detailed information on effects of combined drought and heat stress on yield and physiology of diverse wheat penal, previous studies report effects of combined stresses on only a small set of genotypes. This knowledge, however, could contribute to identifying physiological and biochemical traits tightly involved in heat and drought stress tolerance, useful for wheat genotype selection with improved performance. The present study was aimed.

- To evaluate the effects of combined drought and heat stress on wheat yield and related traits in diverse wheat germplasm.
- To find difference in performance of genotypes with different origin under simultaneous heat and drought stress.
- To explore the role of physio-biochemical traits in simultaneous heat and drought stress tolerance.

## Results

The present study explores the effects of independent and combined drought and heat stress on yield and physiological traits of diverse wheat germplasm. The data was collected from all four controlled environments for two consecutive cropping cycles. Fourteen yield and seven physio-biochemical traits were investigated; the mean values vary considerably across the environments. Detailed information about difference in mean values among different treatment and two cropping season is shown in Table 1.

**Table 1:** Mean values of studied traits for diverse wheat panel planted for two cropping season in control, drought, heat and combination of both heat and drought stress treatment.

Groups	Traits	Control		Drought		Heat		HD	
		2015	2016	2015	2016	2015	2016	2015	2016
Yield Traits	Grains per spike (n)	70.80 ± 0.56	80.09 ± 0.55	59.76 ± 0.65	67.44 ± 0.69	65.84 ± 0.13	69.10 ± 0.22	48.17 ± 0.33	43.09 ± 0.45
	Grain yield (g)	25.07 ± 0.45	26.98 ± 0.77	11.83 ± 0.44	17.04 ± 0.98	10.13 ± 0.45	14.19 ± 0.14	13.88 ± 0.56	9.00 ± 0.14
	Harvest index (%)	51.71 ± 0.74	63.94 ± 0.12	41.63 ± 0.67	30.82 ± 0.22	27.10 ± 0.13	43.11 ± 0.97	37.00 ± 0.89	30.79 ± 0.76
	Spike length (cm)	15.65 ± 1.02	15.57 ± 0.54	14.83 ± 0.44	12.32 ± 0.65	13.86 ± 0.45	9.97 ± 0.71	12.88 ± 0.66	8.85 ± 0.35
	Spikelets per spike (n)	21.45 ± 0.33	23.72 ± 0.11	19.14 ± 0.76	20.07 ± 0.11	18.37 ± 0.45	17.02 ± 0.53	17.73 ± 0.5	18.51 ± 1.05
Phenological traits	Days to anthesis (n)	122 ± 0.16	128 ± 0.32	114 ± 0.66	118 ± 0.08	108 ± 0.76	112 ± 0.10	100 ± 0.67	103 ± 0.43
	Days to maturity (n)	160 ± 0.33	157 ± 0.43	150 ± 0.12	141 ± 0.12	144 ± 0.88	135 ± 0.43	131 ± 0.8	125 ± 0.56
Plant architecture related traits	Awn Length (cm)	6.37 ± 1.11	7.35 ± 0.56	4.9 ± 0.44	3.96 ± 0.34	3.79 ± 0.47	5.60 ± 0.62	3.93 ± 0.4	4.69 ± 0.82
	DW (g)	46.65 ± 0.41	48.87 ± 0.45	39.97 ± 0.12	35.97 ± 0.45	43.51 ± 0.99	37.60 ± 0.37	25.98 ± 0.33	31.56 ± 0.18
	Leaf area	45.54 ± 0.22	49.83 ± 0.13	30.41 ± 0.77	35.11 ± 0.67	35.09 ± 0.67	39.29 ± 0.98	23.43 ± 0.43	26.45 ± 0.13
	Peduncle extrusion (cm)	14.28 ± 0.87	16.78 ± 0.45	10.68 ± 1.03	12.14 ± 0.98	12.81 ± 0.70	8.90 ± 0.45	9.88 ± 0.17	8.44 ± 0.34
	Plant height (cm)	89.38 ± 0.54	93.18 ± 0.88	83.78 ± 1.11	85.15 ± 0.11	76.87 ± 0.98	82.98 ± 0.55	70.92 ± 0.30	62.39 ± 0.13
	Peduncle length (cm)	42.90 ± 0.64	38.80 ± 0.45	35.1 ± 0.98	29.39 ± 0.88	33.71 ± 1.21	33.96 ± 0.88	27.90 ± 0.42	22.24 ± 0.25
	Tillers per plant (n)	7.67 ± 0.89	8.13 ± 0.45	5.73 ± 0.66	7.21 ± 0.53	6.63 ± 0.87	4.62 ± 0.34	3.68 ± 0.45	4.97 ± 0.11

### Analysis of variance and correlation analysis

**Figure 1:**
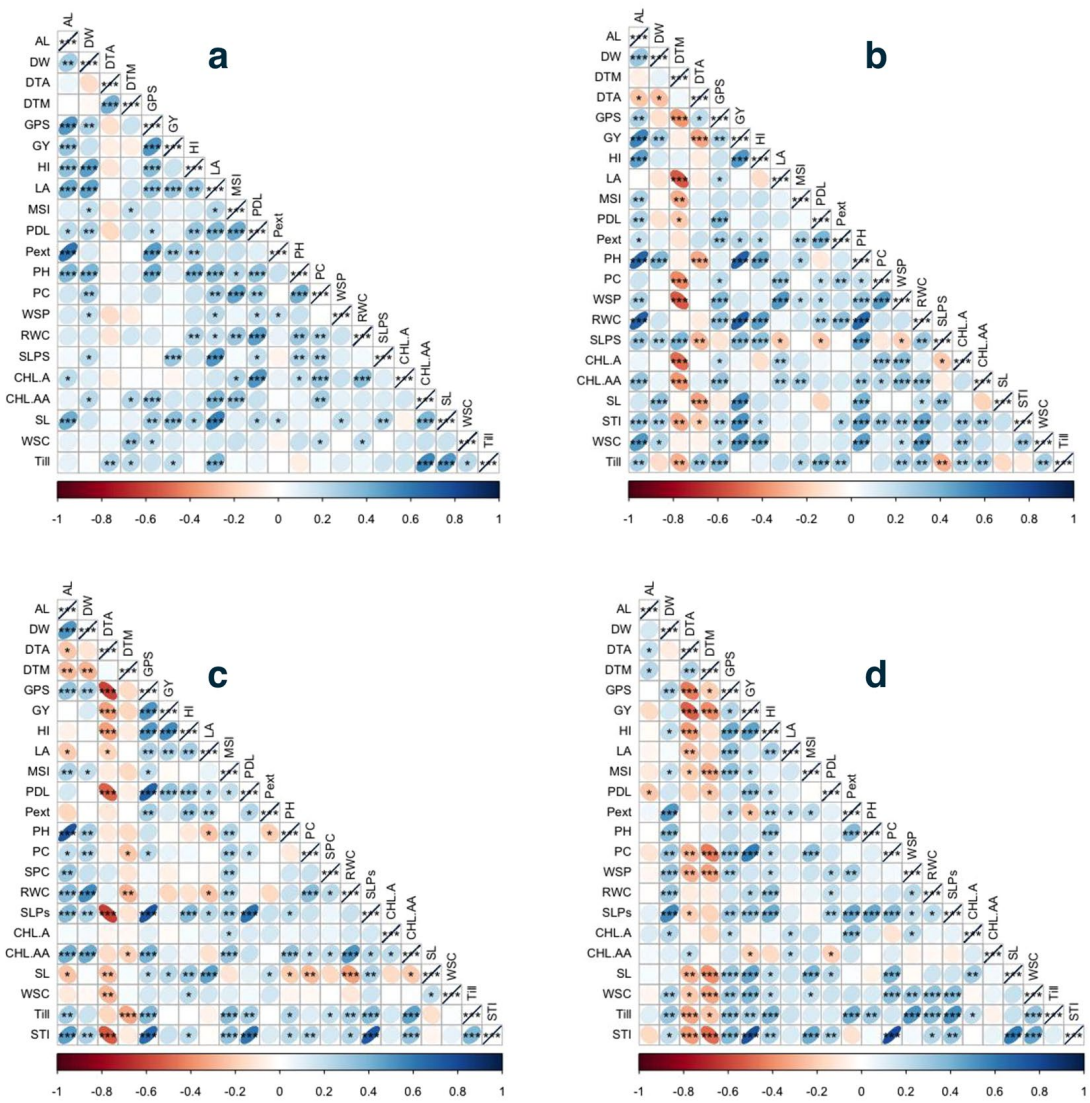
Correlation among studied physiological and morphological traits (**a**) correlation among traits under non stress [C] treatment. (**b**) Correlation among studied traits under drought stress [D] treatment. (**c**) Correlation among studied traits under heat stress [H] treatment. (**d**) Correlation among studied traits under combine heat and drought [HD] stress treatment.

The results from analysis of variance showed significant inhibitory effects of treatments on all studied traits. A significant difference in tolerance of genotypes against stress was also illustrated from analysis of variance (ANOVA) results (Table 2). Under non-stress treatment [C] grain yield had strongest significant correlation with grains per spike (r = 0.51**) followed by spike length (r = 0.34**) while under drought stress [D] GY had highest positive correlation with plant height (r = 0.71**) and relative water content (r = 0.69**). Under heat stress [H] grain yield had strongest correlation with harvest index (r = 0.54**) and grains per spike (r = 0.51**) while under [HD] treatment stress tolerance index (r = 0.76**) and proline content (r = 0.59**) had highest correlation with grain yield. All correlations among yield and physio-biochemical traits are shown in Fig. 1.

**Table 2:** Analysis of variance (ANOVA) table for agronomic traits under glass house conditions.

Traits	Treatment	Genotype	Environment	Treatment: Genotype	Genotype: Environment	Treatment: Environment	Treatment: Genotype: Environment	Residuals
Degree of freedom	3	107	1	321	107	3	321	
Awn Length	16.9**	9.84***	54.25***	2.79***	0.37***	6.39**	0.42***	0.76
Biomass	23282***	207***	350***	48**	17ns	53 ns	15 ns	31
Grains per spike	87569***	785***	11965**	136***	104***	1151***	53**	82
Grain yield	6259***	75***	1653***	22***	13**	586***	12 ns	6
Harvest index	9853***	777***	10907***	208***	119**	5558***	129 ns	101
Leaf area	17282***	743***	480***	50**	55**	13**	7**	34
Peduncle extrusion	40.76***	89.45***	205.59***	26.82**	2.39**	30.44**	1.77 ns	4.79
Peduncle length	665.5***	155.7***	1000***	74.5**	5.3**	112.9***	4.8**	10.4
Plant height	4247***	370**	1380**	100**	15***	116***	8 ns	17
Spike length	2.25***	17.91***	165.01***	3.89***	0.65***	15.74***	0.64***	1.56
Spikelets per spike	70.36***	29.28***	0.1***	8.96 ns	0.06 ns	0.09***	0.24**	1.06
Tillers per plant	888.9***	30.8***	1***	8.4***	1.4***	269.5***	7.2 ns	2.8

**Significant at alpha 0.05.

***Significant at alpha 0.01.

Ns non-significant.

AL: Awn length, DW: Plant above ground biomass, GPS: Grains per spike, HI: Harvest index, LA: Leaf area, PL: Peduncle length, PEXT: Peduncle extrusion, PH: Plant height, SLP: Spikelets per spike, SL: Spike length, TILL: Tillers per plant.

### Effects of treatments on traits

#### Yield-related traits

**Figure 2:**
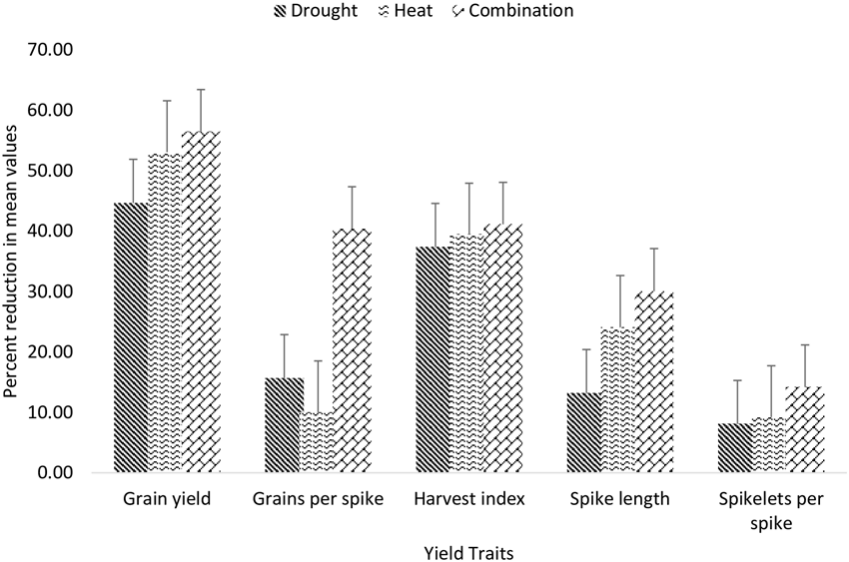
Percent reduction in yield traits of genotypes evaluated under [D], [H] and [HD] stress treatment for two years in glass house conditions.

Among studied yield traits, drought stress had higher reducing effects on grain yield as it caused 45% reductions in mean value. Harvest index was the second most severely affected trait and was reduced by 37% due to an influence of drought while grain per spike was reduced by 16%. Heat stress caused 53% reduction in grain yield followed by 39% reduction in harvest index and 11% percent reduction in grains per spike. When drought and heat stress were imposed simultaneously they caused 56% reduction in grain yield 41% reduction in harvest index and 40% reduction in grains per spike. SLP was reduced by 13% under drought stress while SL was reduced by 12% under drought stress SLP was reduced by 15% while SL was reduced by 24% under heat stress SLP was reduced by 20% while SL was reduced by 30% under combined drought and heat stress (Fig. 2). So in short, among all the studied yield traits GY was severely affected by all three stress treatments followed by HI and GPS. The GPS was more impacted by individual drought stress than individual heat stress while HI and GY were more effected by individual heat stress than individual drought stress. All the yield traits had slightly higher mean values of yield traits during second cropping season than first year (Table 1, Fig. S4). Among the whole panel genotype SAWSN_3059 and SAWSN_3052 performed well under drought stress with higher yield while under heat stress MILLAT-2011 and HTWSN_4427 were to performing genotypes. Two genotypes namely ESWYT_116 and EBWYT_529 had higher values for yield traits under combined heat and drought stress.

#### Phenological traits

**Figure 3:**
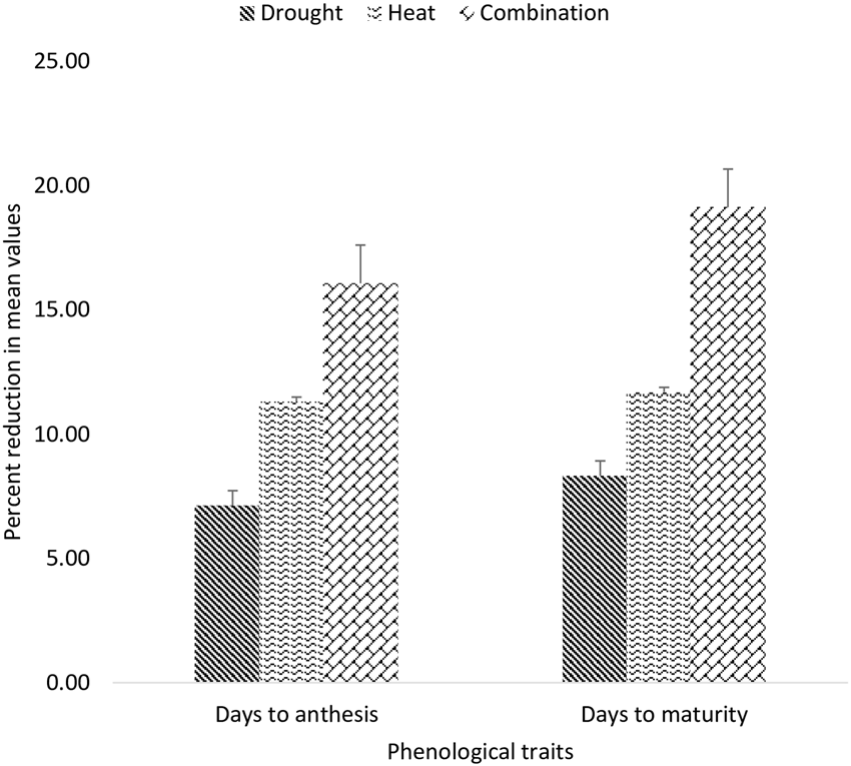
Percent reduction in phenological traits of genotypes evaluated under [D], [H] and [HD] stress treatment for two years in glass house conditions.

Days to anthesis and days to maturity were two phenological traits studied during the present study. Under drought stress days to anthesis and days to maturity were reduced by 10% and 14% while under heat stress these were reduced by 16% and 20% respectively. Combined drought and heat stress caused 25% reduction in DTA and 31% reduction in days to maturity (Fig. 3). Overall, phenological traits were more affected by individual heat than individual drought (Table 1, Fig. S5). Under drought stress genotype SAWSN_3134 had maximum DTA while EBWYT_523 and SRSN_6017 had maximum value for DTA under heat and combined heat and drought stress. Genotype SRSN_6008 under drought stress, ESWYT_110 under heat stress and CHAKWAL-50 under combined drought and heat stress were early maturing genotypes and were favored for further breeding. All these genotypes had least values for DTM among whole studied germplasm.

#### Plant architecture-related traits

**Figure 4:**
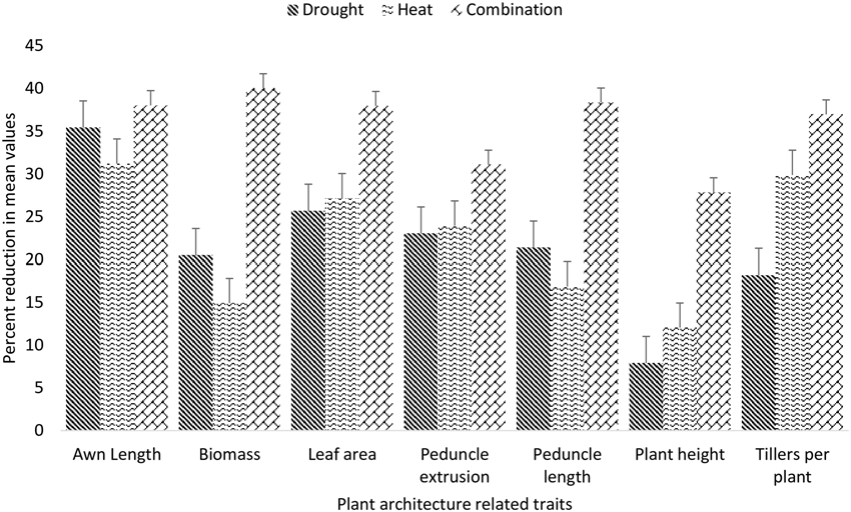
Percent reduction in plant architecture related traits of genotypes evaluated under [D], [H] and [HD] stress treatment for two years in glass house conditions.

Seven plant architecture-related traits were studied during the present study and these included AL, DW, LA, PL, Pext, PH and Till. Under drought stress AL was reduced by 35%, DW was reduced by 21%, LA was reduced by 31%, PL was reduced by 21%, Pext was reduced by 27%, PH was reduced by 7% and Till was reduced by 18%. Heat stress caused 32%, 15%, 22%, 17%, 30%, 12% and 29% reduction in AL, DW, LA, PL, Pext, PH and Till respectively. The combination of both stresses reduced AL by 37%, DW by 40%, LA by 48%, PL by 39%, Pext by 41%, PH by 27% and Till by 45% (Fig. 4). Among these traits, AL, DW and PL were more affected by independent drought stress while remaining traits were affected by individual heat stress. Among whole studied panel EBWYT_524, WYCYT_37, WYCYT_37, ESWYT_107, MILLAT-2011, EBWYT_505 and HTWSN_4421 undergo higher reduction for plant architecture related traits in all stress treatments. All plant architecture related traits have higher mean values during second cropping season except peduncle length (Table 1, Fig. S6).

#### Physio-biochemical traits

**Figure 5:**
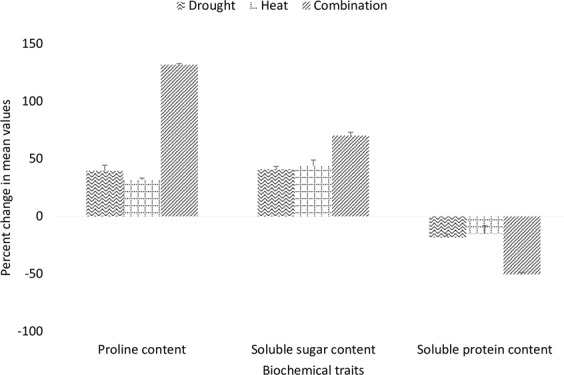
Percent reduction in biochemical traits of genotypes evaluated under [D], [H] and [HD] stress treatment.

Accumulation of compatible solutes in response to the adverse conditions is the first mechanism of adaptation in plants. The present study reports accumulation of proline and water-soluble carbohydrates accumulation in response to drought and heat stress in wheat. An increase of 39, 32 and 132 percent was recorded in mean values of proline content under drought heat and combined drought and heat stress respectively (Fig. 5). Genotype NEPAL-AL_247 had the highest value for proline content under droughts stress while WYCYT_19 and HTWSN_4423 had the highest value under heat stress and the combination of heat and drought stress respectively.

The mean values of genotypes for water-soluble carbohydrates considerably across treatments (Fig. S7). Similar to proline content amount of water-soluble carbohydrates was increased under stress and it was noted that concentration of water-soluble carbohydrates (sugar) was increased by 41%, 44% and 77% under drought, heat and combination of both heat and drought stress respectively (Fig. 5). Among whole studied panel, ESWYT_119 had the highest value of water-soluble carbohydrates under drought stress while EBWYT_511 and NEPAL-AL_247 had highest values under heat and combination of heat and drought stress respectively. Similarly, protein content was reduced by 18% under drought, 15% under heat and 50% by the combination of heat drought stress (Fig. 5).

**Figure 6:**
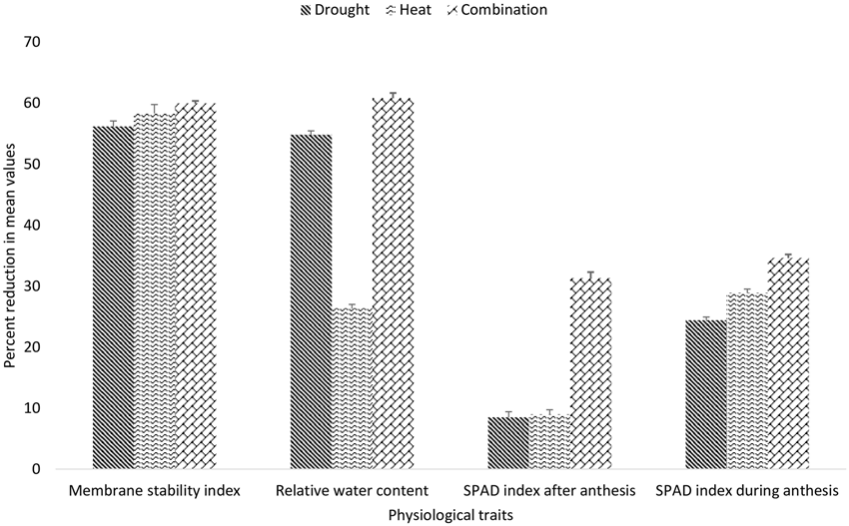
Percent reduction in physiological traits of genotypes evaluated under [D], [H] and [HD] stress treatment.

Mean values for physiological traits varies across the treatments, membrane stability was more affected by high temperature stress while relative water content was more effected by drought stress. Abiotic stresses usually cause disintegration of chlorophyll molecules mostly high temperature is most damaging to the photosynthetic apparatus (Fig. S8). In the present study, chlorophyll content was decreased during and after anthesis and all stress treatments caused a significant reduction in chlorophyll content. During anthesis chlorophyll content was reduced by 24% under drought, 29% under heat and 35% under combined heat and drought stress. Higher reduction in chlorophyll content during anthesis was seen in EBWYT_513 under drought stress and in SRSN_6072 under both heat stress and combined heat and drought stress. The chlorophyll content after anthesis was reduced by 31%, 20% and 8% under combined drought and heat stress, individual drought and individual heat stress respectively (Fig. 6). The higher reduction in chlorophyll content after anthesis was seen in EBWYT_528 under drought stress WYCYT_19 under heat stress and in SAWSN_3087 under combined drought and heat stress.

The relative water content was reduced by 55%, 26% and 61% under drought heat and combined drought and heat stress respectively (Fig. 6). Maximum reduction in RWC was recorded in CHAKWAL-50 under drought stress, EBWYT_519 under heat stress and ESWYT_110 under combined heat and drought stress. Membrane stability index was affected most by combination of drought and heat stress (59.91% reduction) than by heat stress (54.93% reduction) and finally by drought stress (42.59% reduction) (Fig. 6). Among whole panel, ESWYT_131 had the least value for MSI under drought stress while SRSN_6008 and NARC-2011 had the least value under heat and combined heat and drought stress respectively.

### Stress indices

#### Stress tolerance index

Ranking of tolerant genotype was done on the basis of their STI values, under drought stress 53 genotypes had value more than the average value of STI while 55 genotypes had values less than the average value of STI. Under heat stress 60 genotypes had values more than ‘average value of STI while 48 genotypes had values less than average while under combined drought and heat stress 56 genotypes had a higher value than the average STI value and 52 genotypes have least values than STI values. SRSN_6113 and EBWYT_523 have the highest value under combined heat and drought stress, SRSN_6011 and EBWYT_523 had highest values under individual heat stress and ESWYT_117 and WYCYT_16 had the highest values under drought stress.

#### Comparison among different yield nurseries

**Figure 7:**
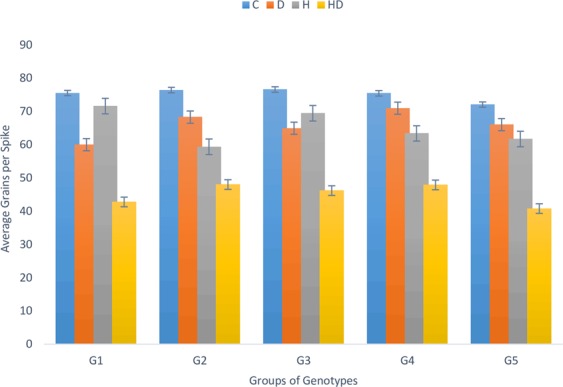
Comparison of grains per spike among different wheat genotype groups under stress and non-stress conditions.

**Figure 8:**
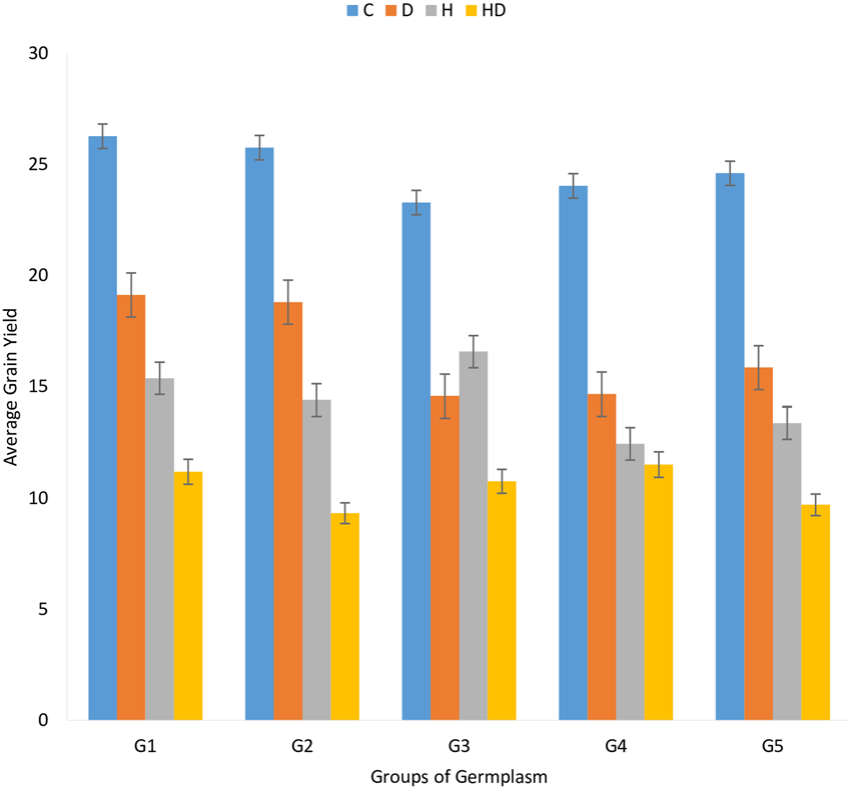
Comparison of grain yield among different wheat genotype groups under stress and non-stress conditions

In the present study advance lines from five CIMMYT nurseries were used to study the effects of independent drought [D], independent heat [H] and combined drought and heat stress [HD]. These nurseries were divided into five major groups the first group (G1) contained members from EBWYT, second group (G2) contained member from (ESWYT) while third group (G3) contained members from (HTWSN + WYCYT + IBWSN). The fourth group (G4) comprise of members from (SAWSN + SRSN) nurseries and fifth (G5) and last group contained local checks (Pakistani varieties). Under optimum conditions group G2 and G3 had maximum grains per spike while local checks have lower average grains per spike under no stress conditions. In drought treatment, members of group G4 had highest grains per spike followed by G2, G5, G3 and G1 respectively. Under heat stress, members of group G1 and G3 had the highest average grain number per spike while members of group G2 and G4 maintained higher kernel number under the combination of heat and drought stress (Fig. 7). G1, G2 and G5 had higher yield under non-stress conditions while members of group G2 had higher yield under drought stress. Group G3 had higher yield under heat stress and group G4 had the highest yield under combined drought and heat stress (Fig. 8).

### Multivariate analysis

**Figure 9:**
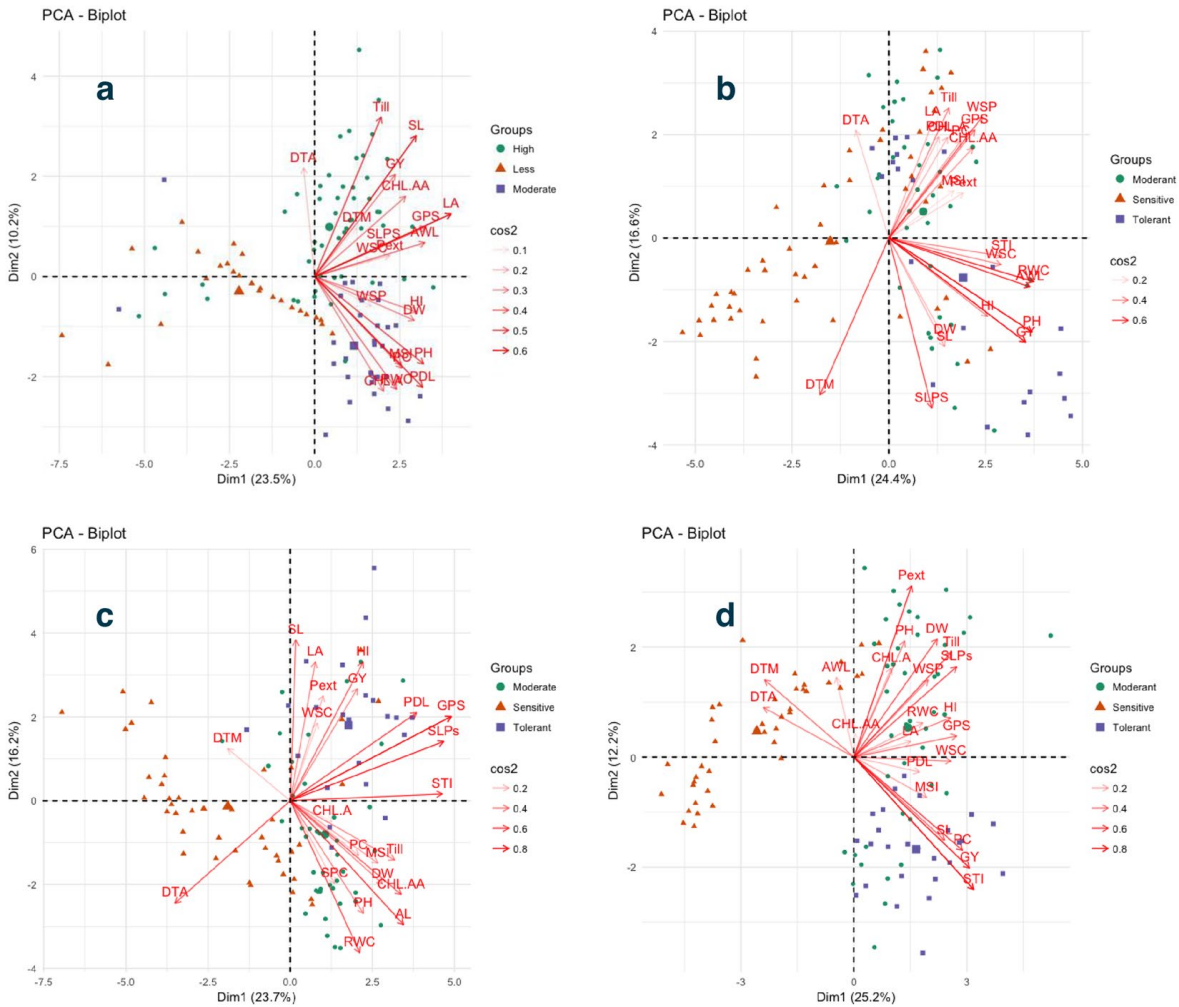
Principal component analysis Biplot. (**a**) PCA biplot for traits and genotypes studied under non [C] stress treatment. (**b**) PCA biplot for traits and genotypes studied under drought [D] stress treatment. (**c**) PCA biplot for traits and genotypes studied under heat [H] stress treatment. (**d**) PCA biplot for traits and genotypes studied under combined [HD] heat and drought stress treatment.

Results from principal component analysis (PCA) demonstrated that under non-stress treatment first seven PCs had Eigenvalues more than 1, first and second principal component explained 23.5 and 10.2% phenotypic variation. The major contributors to these two PCAs were LA, PL, SL, Till, PH, GPS, AL and RWC (Fig. 9a). Under drought stress, first seven PCs had Eigenvalues more than one and first two PCs explained 24.4% and 16.6% phenotypic variation respectively. The major contributors to the first two components under drought stress were PH, GY, RWC, AL, DTM, SLP, WSP and GPS (Fig. 9b). Under heat stress first eight components had Eigenvalues more than one and first two components explained 23.7 and 16.2% phenotypic variation respectively. The major contributors to the first two components were GPS, SLP, STI, AL, PL, DTA, RWC, CHL AA, HI and SL (Fig. 9c). Under combined heat and drought stress, first seven PCs had Eigenvalues more than one, first two components explained 25.2 and 12.2% phenotypic variation respectively. The major contributors to the first two components were STI, GY, Pext, PC, Till, SLP, DW, SL, DTM and GPS (Fig. 9d).

## Discussion

Drought, high temperature and a combination of both, applied at pre-anthesis, decreased physiological and yield traits in hexaploid wheat genotypes and spring wheat cultivars irrespective of the genotype and the time of stress application. Yield traits are considered to be the critical component for enhancing wheat yield, increase in grain yield can be achieved by manipulating yield traits like grains per spike, spike length and spikelets per spike^21^. The main causes of reduction in yield and yield-related traits during stress are pollen abortion^22^, reduction in food reserves^23^ and production of sterile tillers^24^. Reduction in traits depends upon intensity and duration of stress^25^. In the present study drought heat and drought stress caused a significant reduction in traits and independent heat stress had more damaging effect on grain yield than individual drought stress. Many reserve storing traits like plant biomass and leaf area were more affected by independent drought stress than by heat stress. Significant genotype and environment interaction in the present study correspond to the fact that each genotype responded differently to stress treatments. In spite of this, results from stress tolerance index showed that genotypes having higher yield under non-stress [C] treatment showed tolerance (by maintaining their yield) to individual heat and drought and their combination. The significant reduction in wheat yield to individual heat and individual drought and their combination is shown in few studies^6,26,27^.

Stress-generated reactive oxygen species in mitochondria, chloroplast and peroxisome can blow the normal metabolism through oxidative damage of lipids, proteins and nucleic acids^28^ thereby ultimately damaging cell structure^12^. Plants respond to osmotic stress by accumulating compatible solutes such as proline, protein and sugar in the cytosol to balance the osmotic level of cytosol with vacuole and external environment^29^, this protects the cell from injury and maintains the turgor pressure^30,31^. In the present study, higher accumulation of osmolytes is seen under combined drought and heat stress, heat stress caused higher degradation in chlorophyll than individual drought stress. Relative water content was severely affected by drought stress while other all physiological traits were affected by heat stress. The present results are consistent with^32^. Proline ensures successful growth under stress and is involved in various stress signaling pathways which help plant’s adaptation to stress^33,34^. Proline accumulation plays a vital role in combating stress by scavenging Reactive oxygen species (ROS) from the cell without interfering with normal biological processes of cell^35^. Thus protects cell from ROS mediated cellular damage and maintain protein structure inside cell. The results of present study are similar to studies by^36^ on wheat^37^ on rice and^38,39^ on various other crops. Under stressed environments, the photosynthetic ability of the crop decreases thus the amount of carbon reserve for respiration and grain growth also decrease, water-soluble carbohydrate serves as the only source of carbon under these situations^40^. If a genotype had a high reserve of water-soluble carbohydrates than remobilization of these reserves help in maintaining grain yield and enhance tolerance of crop to stress^40–42^.

Changes in the structure of chloroplast i.e. change in shape of chloroplasts, swelling of stromal lamellae, clumpy vacuoles, antenna-depleted PS II and degradation of chlorophyll molecules during stress results in the reduction in chlorophyll content^43–45^. Furthermore, heat stress cause more damage to photosynthetic electron transport chain^46^ and degrade enzyme involved in photosynthesis^47^ than drought stress. In the present study, the pre-anthesis and post-anthesis chlorophyll content were severely affected by individual heat and combined drought and heat stress. Relative water content is an indicator of water status of the plant any reduction in RWC cause loss of turgidity which in turn affects cell size and shape of plants^48^. In addition, reduced water content induces photorespiration due to closure of stomata which generates reactive oxygen species involved in various cellular damages^49^. In the present study water content was more affected under drought stress than heat and results are in agreement with^45^. Accumulation of protein stress is necessary for maintaining osmotic balance^50,51^ and membrane stability^52^ under stressed environment.

## Conclusion

In conclusion, drought and stress alone and in combination reduced grain yield and its components, grain yield was most affected under individual heat stress while grain number was more reduced by individual drought stress than individual heat stress. Among physiological traits, plasma membrane stability was highly affected by individual heat stress and relative water content was severely affected by individual heat stress. Genotype which had higher yield under stress were found to be tolerant under stress conditions this illustrates the capacity of wheat genotypes to capitalize improved environmental conditions late during reproductive stage through GY modifications in response to compensatory mechanisms. The principal component analysis highlighted many of the included physiological and biochemical parameters late in the reproductive stage as powerful explanatory variables of yield and its components variations, suggesting that they might be useful for cultivar screening. Improvement of trait having a major contribution in the first two components may lead to improved varieties with heat and drought stress tolerance. The present study is a first detailed and comprehensive study involving diverse germplasm to understand the combined effects of drought and heat stress under controlled conditions. Further studies are necessary involving more diverse genotypes and years to validate the potential power of the mentioned selection markers for heat and water-stressed wheat.

## Methods

### Plant materials

One hundred and eight advance wheat lines from CIMMYT heat and drought nurseries previously screened for high performance under rain-fed conditions were used in the present study. The germplasm consisted of 98 advanced lines from CIMMYT and ten local high yielding varieties were used to compare yield among international and local varieties.

### Experimental and treatment conditions

Seeds of all genotypes were obtained from the Wheat Wide Crosses Laboratory, National Agriculture Research Center (NARC) Islamabad, Pakistan. Six seeds of each genotype were sown in plastic pots (30 cm in diameter, 40 cm in depth) filled with loamy soil (80% sand, 15% silt and 5% clay) and after thinning three plants of each genotype were kept with nine replications for each genotype and maintained at normal conditions till heading with all normal agronomic practices. After heading the pots were shifted to glass house for stress treatments and the treatments were imposed from heading till maturity: Drought [D] plants were shifted in glass house with normal day/ night (21/15 °C) temperature and moisture content was maintained at 30% field capacity; Heat [H] plants were shifted after heading to a glass house with 36/30 °C day/night temperature and normal irrigation and a combination of heat and drought stress [D + H] plants were shifted after heading to a glass house with 36/30 °C day/night temperature and moisture content was maintained at 30% field capacity. One complete set was grown as non-stress treatment [C] normal irrigation with 25/15 °C day/ night temperature. Temperature and precipitation varied considerably during two years at the experimental location (Figs S1 and S2). For drought treatment, the moisture content of pots was maintained at 30% of the total available water capacity using Time Domain Reflectometry (TDR). When the moisture content of the pot was declined to less than 30% of the total available water capacity of the soil pots were re-watered with 300 ml of water to avoid permanent wilting (Fig. S3). The stress period was from heading till maturity. In our experiments, we were interested in drought stress resulting from soil water deficit. The relative humidity of the growth chamber was kept high by wetting the floor and by turning on evaporative cooling pads, to prevent drought stress due to rapid evapotranspiration under the elevated air temperature. The rain fed wheat areas of the world may not have such a high relative humidity at the reproductive stages. After stress treatment, plants were moved back to optimum condition. Every alternate day, plants in each growth chamber were randomly moved to avoid any positional effect within the chamber. Nutrient supply to plants was provided by adding water-soluble Phosphatic fertilizer like DAP and SONA Urea contains 46% P_2_O_5_ and 46% Nitrogen respectively, to pots three times during germination to heading and once before the start of stress treatments. The same experiment was repeated for two cropping seasons, i.e. 2015 and 2016.

### Data collection

The yield traits were recorded from all three plants per genotype while physiological data was recorded from three randomly selected plants. Following yield traits were recorded during study; awn length (AL) (from tip of spike), days to anthesis (DTA) (number of days taken from emergence to appearance of anthers), day to maturity (DTM) (number of days taken from emergence to maturity), grains per spike (GPS) (number of grains in spike was counted), grain yield (GY) (weighing grains form all harvested plants), harvest index (HI) (percent ratio of grain yield and above ground plant dry weight (DW), peduncle extrusion (PEXT) (from tip of flag leaf to base of spike), peduncle length (PL) (from first node to base of spike), plant height (PH) (from ground level to spike tip excluding awns), spikelet number (SLP) (counting number of fertile tillers), spike length (SL) (manually with ruler in cm).

For physiological and biochemical studies, the data was recorded using standard protocols Chlorophyll Content (SPAD index).

Flag leaf chlorophyll content was determined during and after anthesis using a SPAD 502 (Minolta Spectrum Technologies Inc., Plainfield, IL, USA) portable leaf chlorophyll meter^53^. Three readings from a single flag leaf were taken and averaged and in the same way data was recorded form six flag leaves from a single pot.

#### Water soluble carbohydrates in leaves (WSC)

Fresh flag leaves (0.1 g) were added with 5 ml of 80% ethanol to test tubes, placed and heated in water bath for 1 hour at 80 °C. Then, 1 ml of the sample extract was taken in another set of test tubes and mixed with 1 ml each of 18% phenol and distilled water, and then allowed to stand at room temperature for an hour. Finally, 5 ml of Sulphuric acid was added and the whole mixture was vortexed. The absorbance was read at 490 nm wavelength on the UV spectrophotometer (UV/Vis Scanning Models/UV-3200(PC). Ethanol 80% was used as blank sample^54^.

$${\rm{Water}}\,{\rm{soluble}}\,{\rm{carbohydrates}}=\frac{{\rm{Absorbance}}\,{\rm{of}}\,{\rm{Sample}}\times {\rm{K}}\,{\rm{value}}\times {\rm{Dilution}}\,{\rm{factor}}}{{\rm{Weight}}\,{\rm{of}}\,{\rm{sample}}\times 100}$$

#### Proline content (PC)

The proline content was estimated by using method developed by. Briefly, 0.1 g fresh leaf sample was ground in 5 ml of 3% sulfosalicylic acid and then allowed to settle for two hours. After 2 hours 2 ml of supernatant was taken and 2 ml of both glacial acetic acid ninhydrin reagent were added to reaction mixture and whole mixture was boiled in water bath for 1 hr at 100 °C. After completion of reaction the test tubes were taken out from water bath and allowed to cool at room temperature. The procedure was ended by addition of 4 ml of Toluene to reaction mixture after short vortex and absorbance was read at 520 nm on UV Spectrophotometer (UV/Vis Scanning Models/UV-3200(PC)). Toluene was used as blank sample^55^.

$${\rm{Proline}}\,{\rm{Content}}=\frac{{\rm{Absorbance}}\,{\rm{of}}\,{\rm{Sample}}\times {\rm{K}}\,{\rm{value}}\times {\rm{Dilution}}\,{\rm{factor}}}{{\rm{Weight}}\,{\rm{of}}\,{\rm{sample}}\times 100}$$

#### Soluble protein content (WSP)

Protein content was determined by using Bomine serum albumin (BSA) as standard (Fresh)^56^. Fresh leaves (0.1 g) were grounded in 5 ml phosphate buffer with the help of pestle and mortar around ice. 0.5 ml of extract and distilled water were added to test tube and along with 3 ml of bio-red color dye and whole mixture was vortexed for a while. The absorbance was recorded at 595 nm on the UV Spectrophotometer (UV/Vis Scanning Models/UV 3200(PC)). Phosphate buffer was used as a blank sample.

$${\rm{Total}}\,{\rm{Soluble}}\,{\rm{Protein}}=\frac{{\rm{Absorbance}}\,{\rm{of}}\,{\rm{Sample}}\times {\rm{K}}\,{\rm{value}}\times {\rm{Dilution}}\,{\rm{factor}}}{{\rm{Weight}}\,{\rm{of}}\,{\rm{sample}}\times 100}$$

#### Relative water content (RWC)

Fresh, mature and fully extended leaves were cut from three random plants and immediately placed in ice box. The flag leaves were cut unto 5 cm long fragments and fresh weight was taken immediately. Leaves were than soaked in distilled water for 24 hours and after 24 hours turgid weight was recorded. After that leaves were kept in oven at 80 °C for 24 hours to record dry weight. The relative water content was recorded using following formula^57^.

$${\rm{Relative}}\,{\rm{Water}}\,{\rm{Content}}=\frac{{\rm{Fresh}}\,{\rm{Weight}}-{\rm{Dry}}\,{\rm{Weight}}}{{\rm{Turgid}}\,{\rm{Weight}}-{\rm{Dry}}\,{\rm{Weight}}\,({\rm{g}})}\times 100$$

#### Membrane stability index (MSI)

Three mature flag leaves were randomly taken from each treatment and were chopped into 3.5 cm long pieces. After washing, two sets of test tubes were made each containing 10 ml of water and a piece of flag leaf. One set was regarded as control and other were regarded as Treatment. The treatment set of test tubes was wrapped with paraffin film and heated in water bath at 45 °C for 1 hour (T1) while control was kept at room temperature (25 °C). The tubes were kept at 10 °C for 24 hours to allow leakage of electrolytes form leaves. After 24 hours tubes were shifted to room temperature shaken well and electric conductivity (C1) was recorded. The tubes were than heated at 100 C for 30 minutes (T2) to release all electrolytes and then cooled at room temperature. After shaking the final electric conductance was measured (C2). Membrane stability index was expressed as percent relative injury (RI%) using following expression^58^.

$${\rm{CMS}}\,( \% )=1-\frac{[1-({\rm{T}}1/{\rm{T}}2)}{1-(\frac{{\rm{C}}1}{{\rm{C}}2})}\times 100$$

#### Stress tolerance index (STI)

Stress tolerance index was used determine tolerance of genotypes to stress by using following formula^59^.

$${\rm{Stress}}\,{\rm{tolerance}}\,{\rm{index}}\,({\rm{STI}})=\frac{{\rm{Yp}}-{\rm{Ys}}}{\bar{Y}{p}^{2}}\times 100$$

Ys is yield of each genotype under non-stress environment.

Yp represents yield of each genotype in stressed conditions.

$\bar{Y}{p}^{2}$ is yield means in non-stress conditions for all genotypes.

### Statistical analysis

The significance of differences between treatments was analysed using analysis of variance (ANOVA) techniques appropriate for factorial design according to a complete randomized design (CRD). The effect of treatment, genotype and environment and significance of differences between treatments means (Tukey’s test) was determined by using “*Agricolae*” package in R. The summary statistic and correlation among traits performed in R using mean value for each genotype. The relationship among yield and physiological traits was further explored using principal component analysis technique. Results of PCA were visualized using Biplot constructed between first two principal components (PC1 and PC2) using R packages^60^.

## Supplementary information

Supplementary information
