# Supplementary material for: Effects of Pre-Anthesis Drought, Heat and Their Combination on the Growth, Yield and Physiology of diverse Wheat (Triticum aestivum L.) Genotypes Varying in Sensitivity to Heat and drought stress
Source: Sci Rep. 2019 May 6;9:6955. doi: 10.1038/s41598-019-43477-z (PMC6502848; doi:10.1038/s41598-019-43477-z)
Supplement: Supplementary file 1 — Supplementary information [file 41598_2019_43477_MOESM1_ESM.pdf]

Effects of Pre-Anthesis Drought, Heat and Their Combination on the Growth, Yield and Physiology of diverse Wheat (*Triticum aestivum* L.) Genotypes Varying in Sensitivity to Heat and drought stress.

Mirza Faisal Qaseem[1*] Rahmatullah Qureshi[1]and Humaira Shaheen[2]

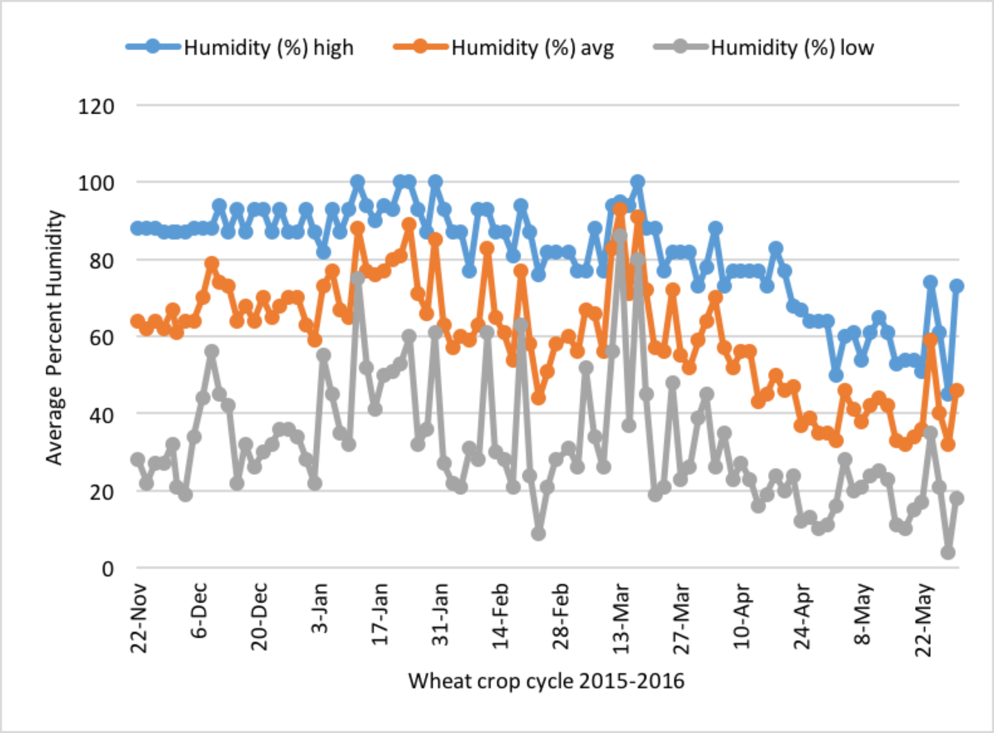

Figure S1. Minimum, Maximum and average temperature of trial location during two cropping seasons 2015-2016

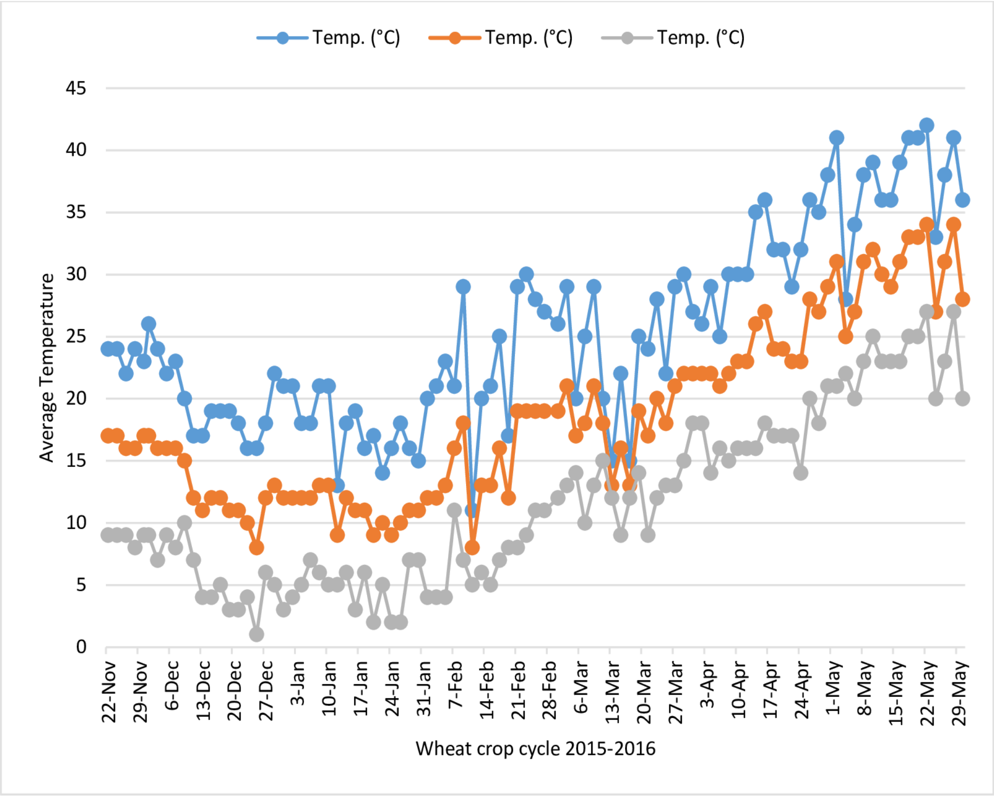

Figure S2. Minimum, Maximum and average precipitation of trial location during two cropping seasons 2015-2016

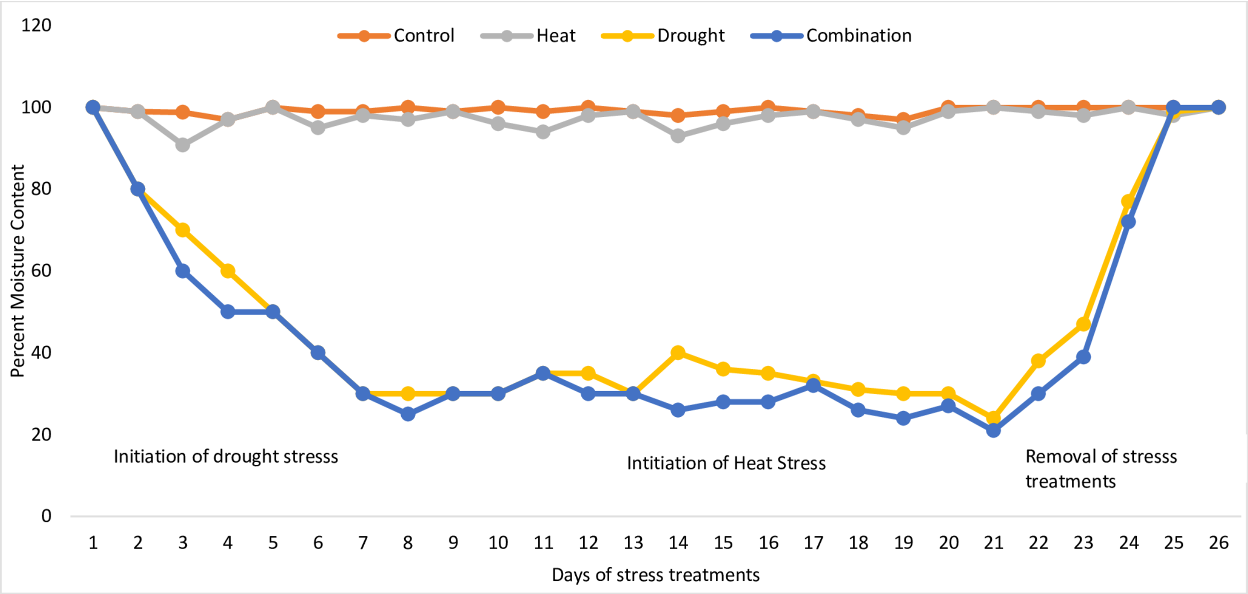

Figure S3. Soil moisture content during the drought treatment, heat treatment period and post-stress period calculated as the mean of the pots planted with the three plants

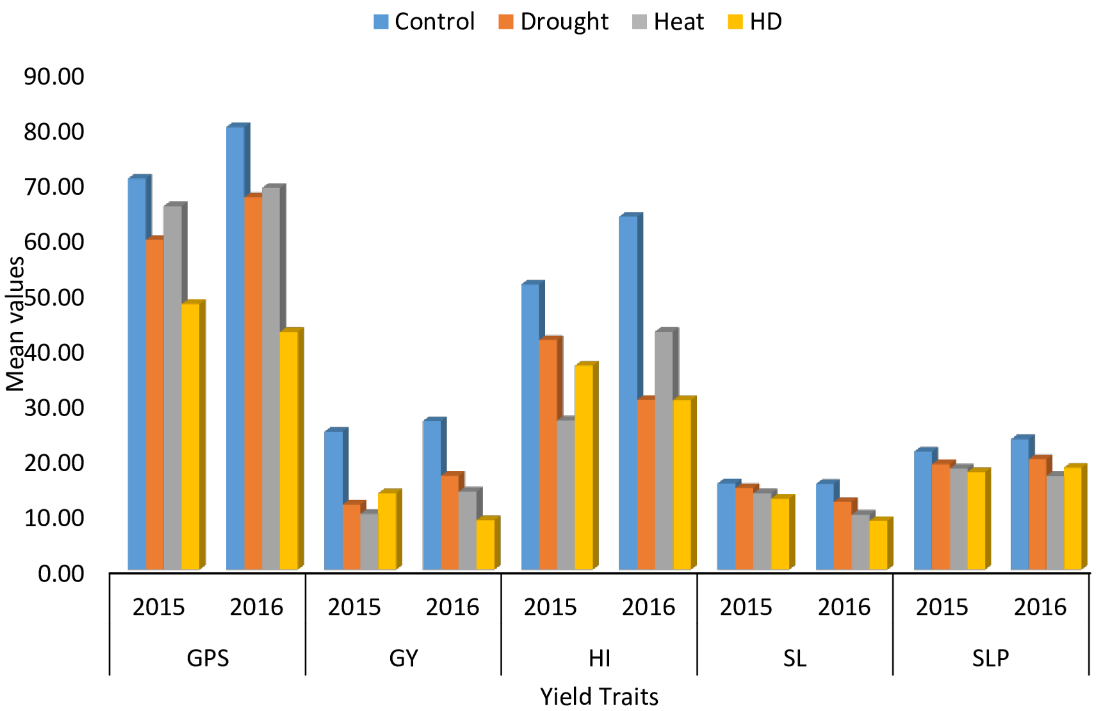

Figure S4: Change in mean values of yield traits across the two cropping years and four stress treatments i.e. [C], [D], [H] and [HD].

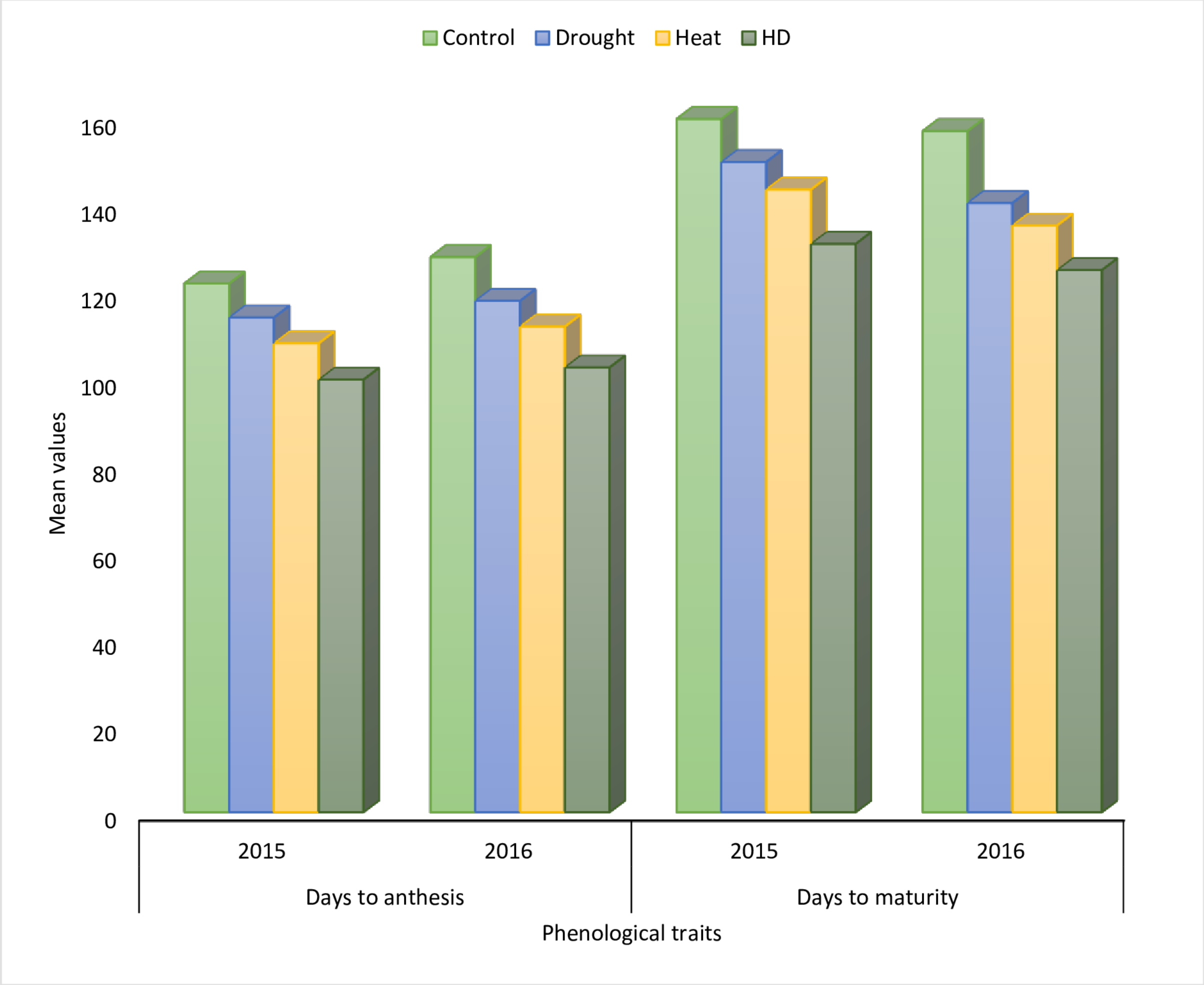

Figure S5: Change in mean values of Phenological traits across the two cropping years and four stress treatments i.e. [C], [D], [H] and [HD].

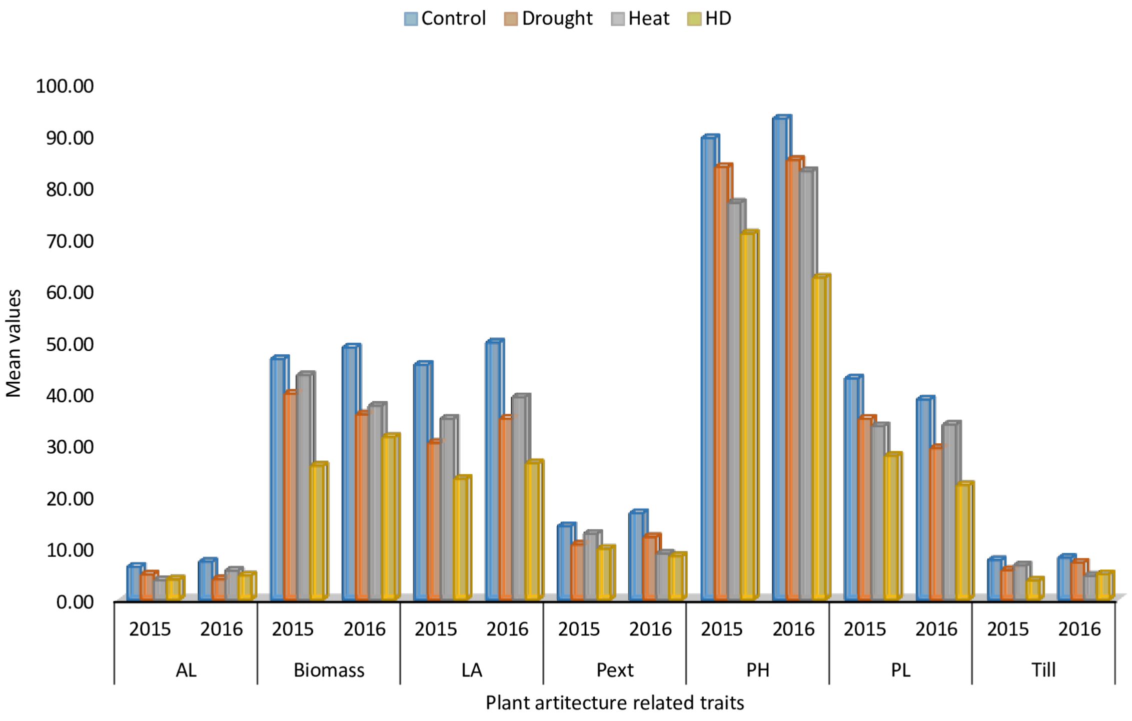

Figure S6: Change in mean values of plant architecture related traits across the two cropping years and four stress treatments i.e. [C], [D], [H] and [HD].

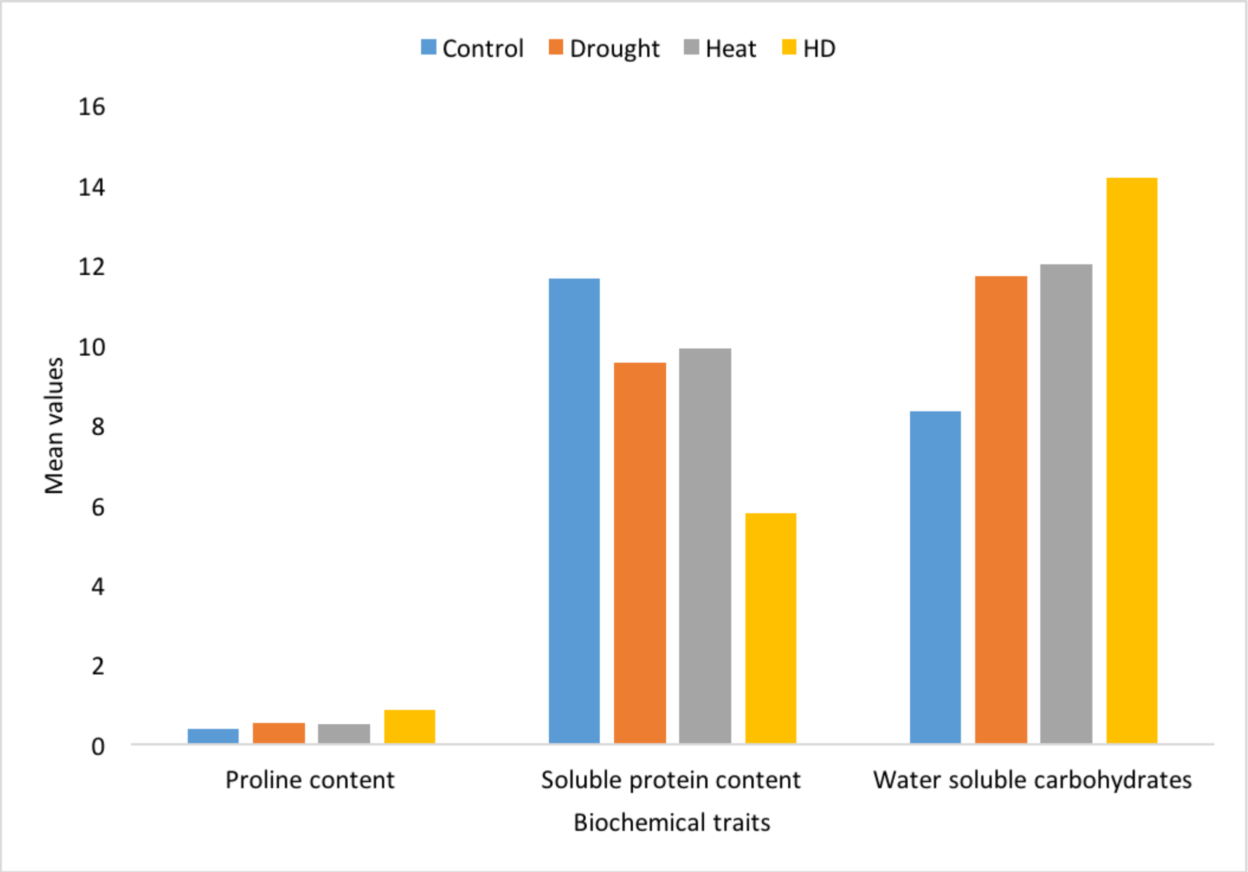

Figure S7: Change in mean values of Biochemical traits against four stress treatments i.e. [C], [D], [H] and [HD].

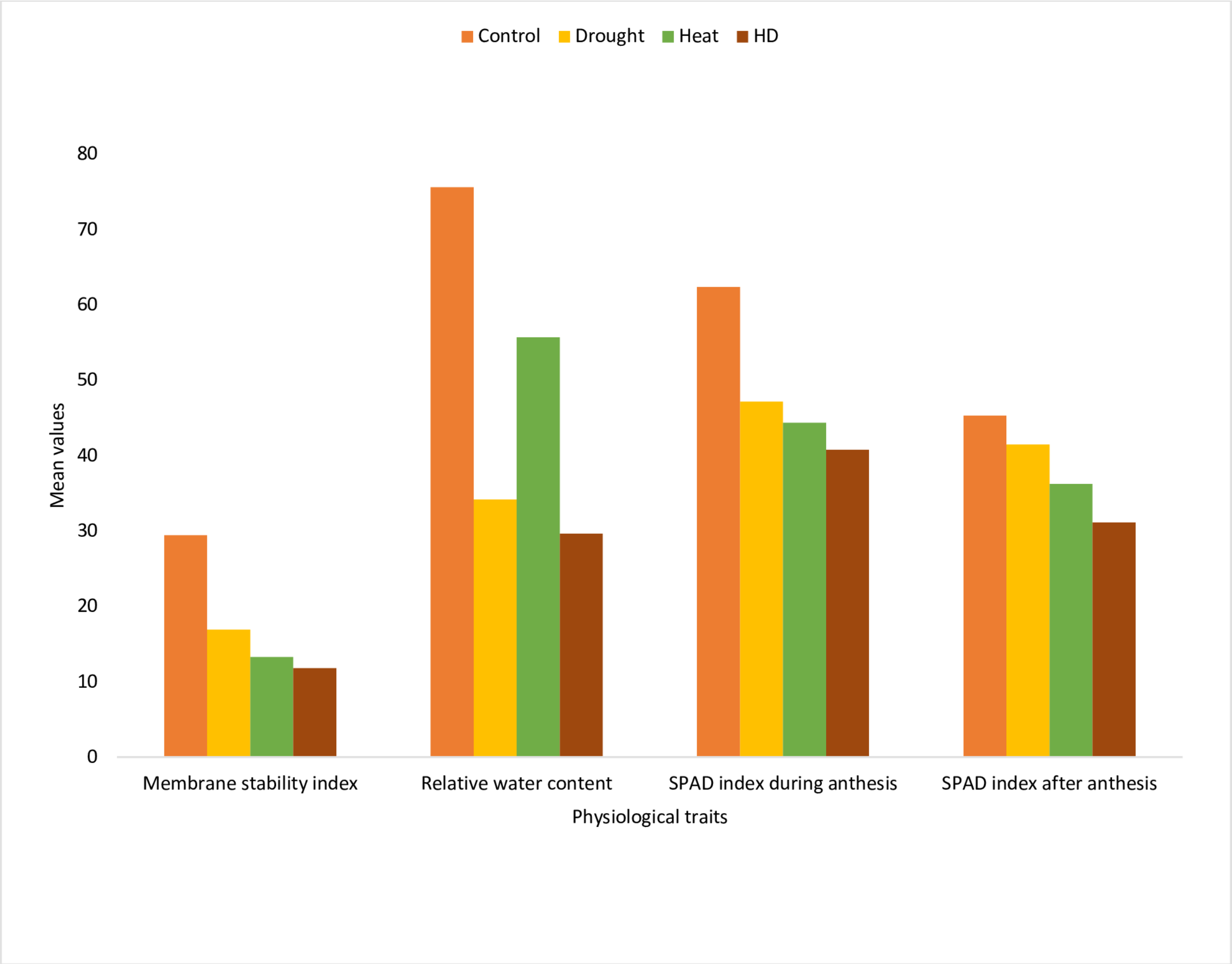

Figure S8: Change in mean values of physiological traits against four stress treatments i.e. [C], [D], [H] and [HD].

Appendix 1: Effect of Heat and drought stress on agro-physiological traits   C: Control D: Drought, H: Heat, HD Combined Drought and Heat stress
Alphabetic letters: LSD groups

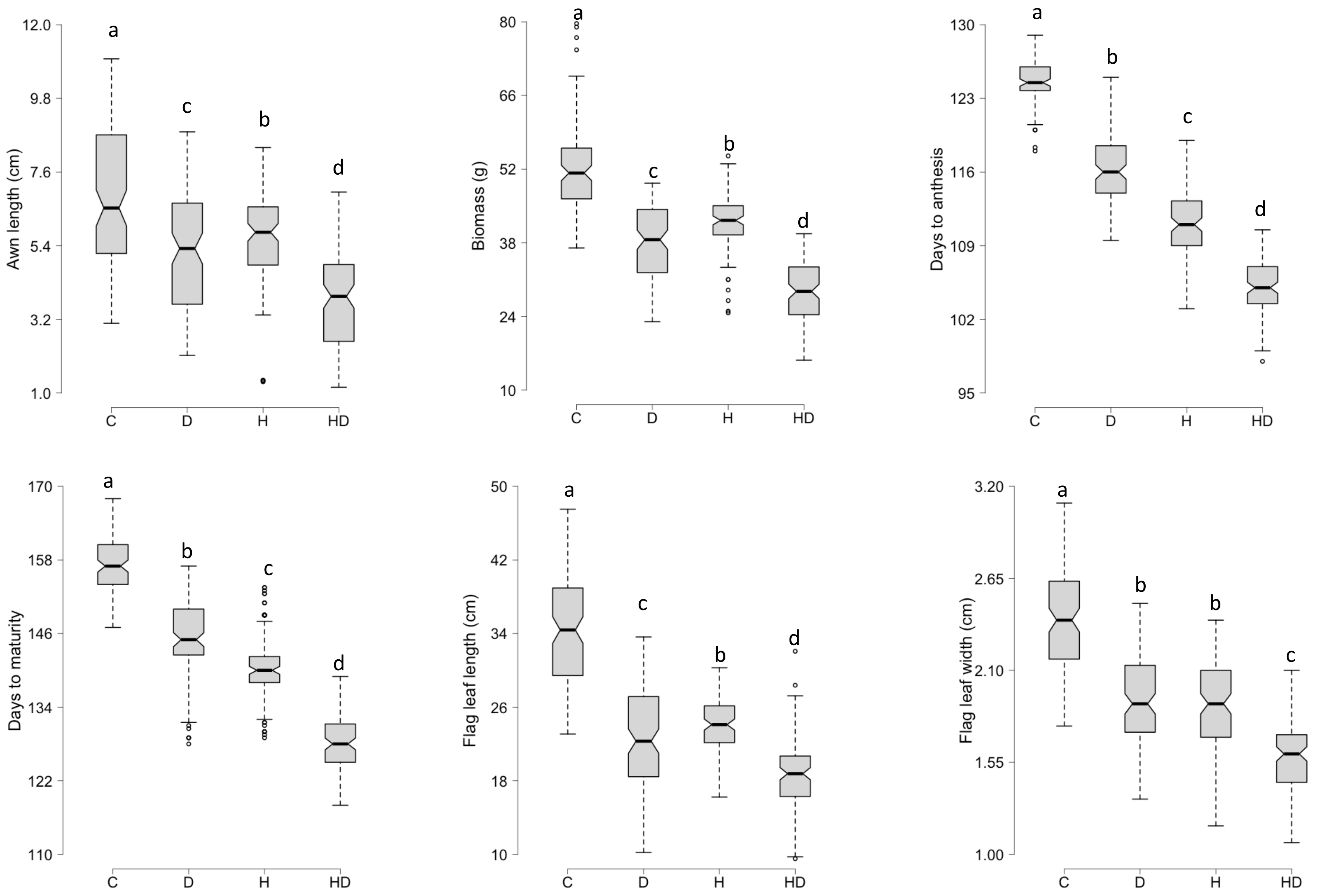

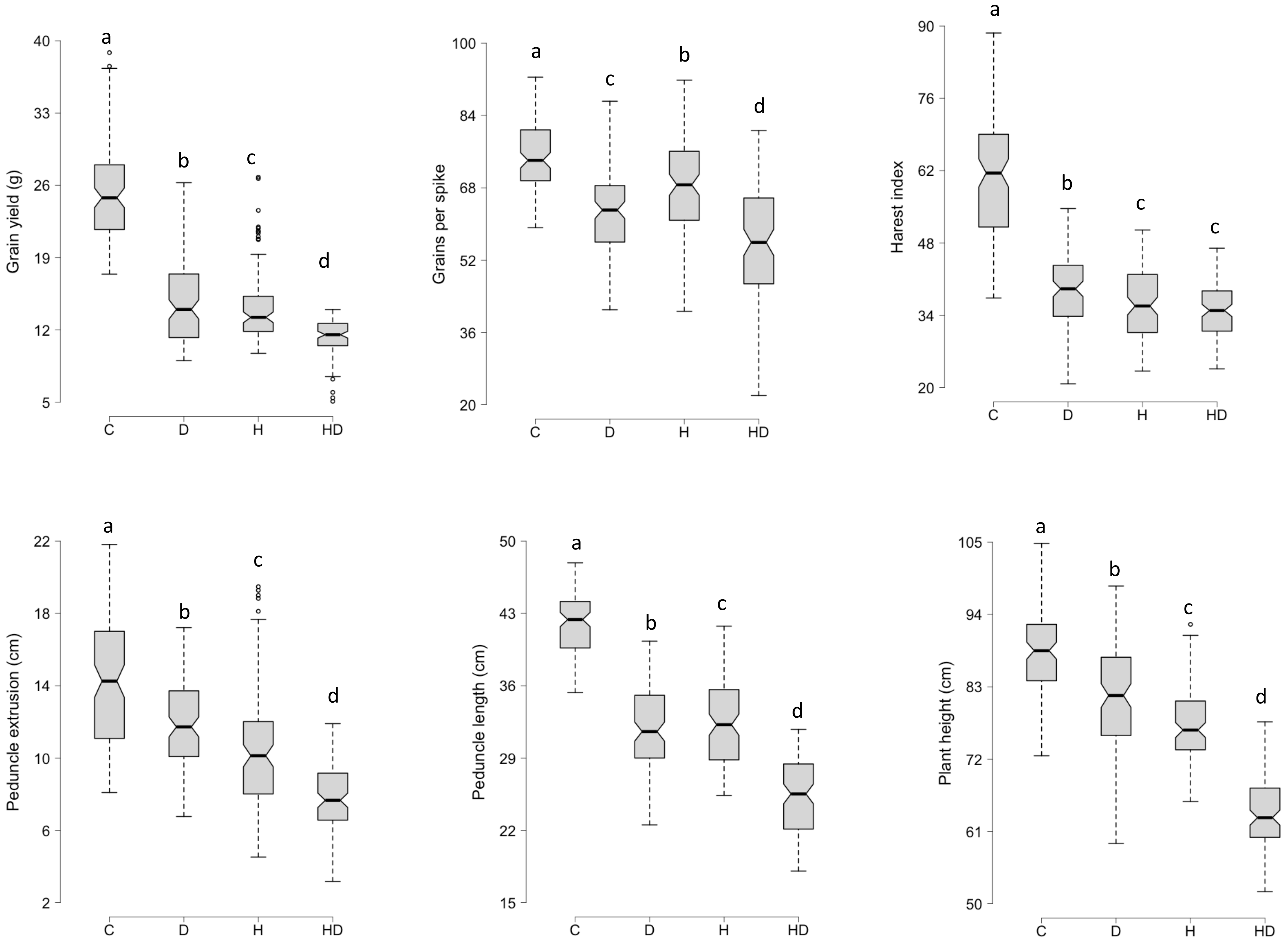

Grain yield (g)
Grains per spike
Harest index
Peduncle extrusion (cm)
Peduncle length (cm)
Plant height (cm)
C
D
H
HD
a
b
c
d

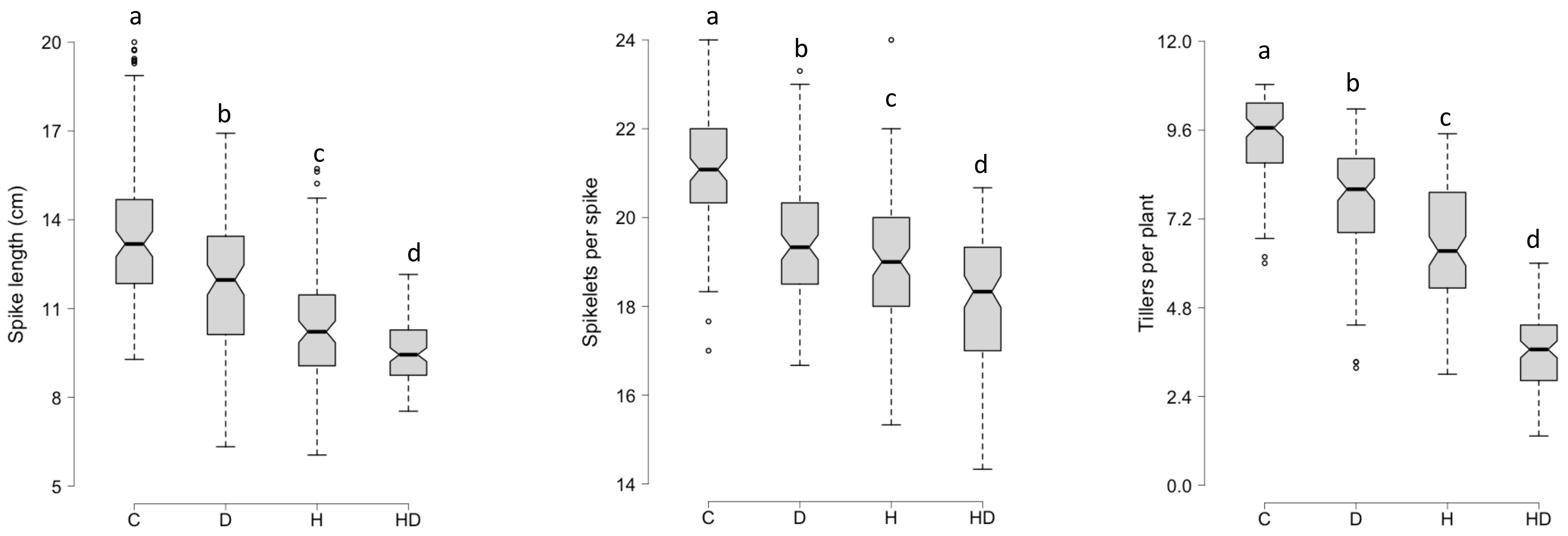
Spike length (cm)
20
17
14
11
8
5
C
D
H
HD
a
b
c
d
Spikelets per spike
24
22
20
18
16
14
C
D
H
HD
a
b
c
d
Tillers per plant
12.0
9.6
7.2
4.8
2.4
0.0
C
D
H
HD
a
b
c
d

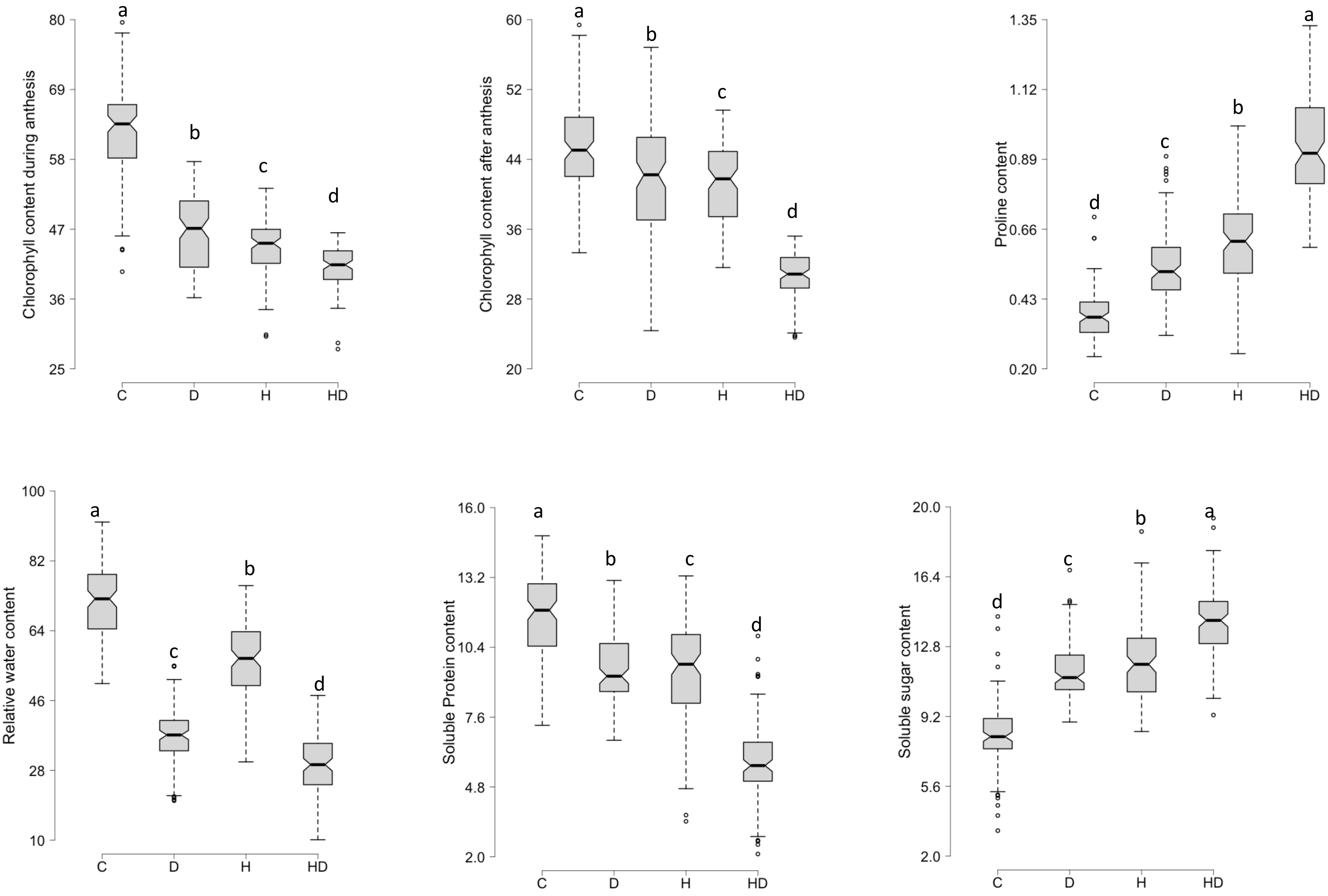

Chlorophyll content during anthesis
80
69
58
47
36
25
C
D
H
HD
a
b
c
d
Chlorophyll content after anthesis
60
52
44
36
28
20
C
D
H
HD
a
b
c
d
Proline content
1.35
1.12
0.89
0.66
0.43
0.20
C
D
H
HD
d
c
b
a
Relative water content
100
82
64
46
28
10
C
D
H
HD
a
c
b
d
Soluble Protein content
16.0
13.2
10.4
7.6
4.8
2.0
C
D
H
HD
a
b
c
d
Soluble sugar content
20.0
16.4
12.8
9.2
5.6
2.0
C
D
H
HD
d
c
b
a
